# Neural representation of linguistic feature hierarchy reflects second-language proficiency

**DOI:** 10.1016/j.neuroimage.2020.117586

**Published:** 2020-12-24

**Authors:** Giovanni M. Di Liberto, Jingping Nie, Jeremy Yeaton, Bahar Khalighinejad, Shihab A. Shamma, Nima Mesgarani

**Affiliations:** a Laboratoire des systèmes perceptifs, Département d’études cognitives, École normale supérieure, PSL University, CNRS, 75005 Paris, France; b Department of Electrical Engineering, Columbia University, New York, NY, USA; c Mortimer B. Zuckerman Mind Brain Behavior Institute, Columbia University, New York, NY 10027, United States; d Laboratoire de Psychologie Cognitive, UMR 7290, CNRS, France. Aix-Marseille Université, France; e Institute for Systems Research, Electrical and Computer Engineering, University of Maryland, College Park, USA

## Abstract

Acquiring a new language requires individuals to simultaneously and gradually learn linguistic attributes on multiple levels. Here, we investigated how this learning process changes the neural encoding of natural speech by assessing the encoding of the linguistic feature hierarchy in second-language listeners. Electroencephalography (EEG) signals were recorded from native Mandarin speakers with varied English proficiency and from native English speakers while they listened to audio-stories in English. We measured the temporal response functions (TRFs) for acoustic, phonemic, phonotactic, and semantic features in individual participants and found a main effect of proficiency on linguistic encoding. This effect of second-language proficiency was particularly prominent on the neural encoding of phonemes, showing stronger encoding of “new” phonemic contrasts (i.e., English contrasts that do not exist in Mandarin) with increasing proficiency. Overall, we found that the nonnative listeners with higher proficiency levels had a linguistic feature representation more similar to that of native listeners, which enabled the accurate decoding of language proficiency. This result advances our understanding of the cortical processing of linguistic information in second-language learners and provides an objective measure of language proficiency.

## 1. Introduction

Learning a second language (L2) via instruction is a time-consuming and challenging process. Adult learners rarely attain native-like L2 proficiency and instead carry-over features of their native languages (L1) to their L2 (Hartshorne et al., 2018; Kotz, 2009; Kuhl, 2011), which can have a major impact on their social lives (DeJesus et al., 2017; Lev-Ari and Keysar, 2010; Mayo et al., 1997). Despite a considerable amount of literature, there remains considerable uncertainty regarding the precise neural changes that underpin the increased L2 proficiency that develops during the learning process (Bohn and Munro, 2007; Marsden et al., 2013; Perani and Abutalebi, 2005). Studies involving hemodynamic measurements (e.g., fMRI) have provided us with precise insights into the sources of the cortical activations corresponding to L1 and L2 processing while substantially neglecting the rich temporal dynamics of speech. This limitation is a likely cause of the apparent inconsistency between studies showing cortical activation for L1 and L2 processing in the same areas (Chee et al., 1999; Nakada et al., 2001; Perani and Abutalebi, 2005; Tan et al., 2003; Yokoyama et al., 2006) or in both shared and distinct areas (Chee et al., 2001; Kim et al., 1997; Xu et al., 2017; Xue et al., 2004). Studies based on electrical neural recordings, such as electro and magneto-encephalography (EEG and MEG respectively), provide a different view of this issue, showing delayed lexico-semantic processing for the less proficient L2 compared to that for L1 (Hahne, 2001; Phillips et al., 2006). More recently, studies with both high temporal and spatial detail (based on multimodal imaging approaches) suggested that acquiring a language may involve the recruitment of cortical areas that are not necessary once fluency is achieved (Leonard et al., 2010).

To elucidate the neural mechanisms that underlie L2 perception, it is crucial to assess the effect of proficiency on objective neural measures that capture the multifaceted cortical encoding of language. This is a complex task, especially because speech perception involves the analysis of various acoustic and linguistic features, a process that is thought to engage a hierarchical neural network composed of various interconnected cortical regions (Hickok and Poeppel, 2007). Distinct stages of processing have been shown to be affected differently by proficiency, with some of the stages becoming more those observed for the native language than other stages in proficient L2 users. Part of the evidence comes from EEG and MEG research, which showed the effect of proficiency at the levels of phonemes (White et al., 2012), syntax (Hanna et al., 2016; Weber-Fox and Neville, 1996), and semantics (Ojima et al., 2005). These studies measured the changes in well-known event-related potential (ERP) components, such as the MMN, N400, and P600. These approaches, however, use unnatural speech stimuli (e.g., isolated syllables or violative speech sentences) that do not fully and realistically activate the specialized speech cortex (Overath et al., 2015; Van Petten and Kutas, 1990; Zinszer et al., 2015). In addition, these approaches consider various levels of speech perception independently and in isolation. Language learning, on the other hand, involves the simultaneous acquisition of novel phonetic contrasts (Chomsky and Halle, 1968; Ladefoged and Johnson, 2014), new syllabic structures (phonotactics) (Trubetzkoy, 1969), and new words. A more complete view of the neural basis of language learning therefore requires a joint study of multiple levels of the linguistic hierarchy to advance our understanding of L2 perception by informing us of the precise effect of proficiency on the cortical processing strategies that underpin sound and language perception (Federmeier, 2007; Huettig and Mani, 2015; Kuperberg and Jaeger, 2016).

Previous efforts in using naturalistic speech stimuli to study language proficiency showed a modulation of EEG phase synchronization while listening to naturalistic speech at both the subcortical (FFR; Krizman et al., 2015; Omote et al., 2017) and cortical levels (gamma EEG synchrony; Reiterer et al., 2011, 2009). Specifically, stronger synchrony between EEG channels was shown for low-proficiency users (Reiterer et al., 2011), which is in line with theories, such as the *cortical efficiency theory*, positing that less experienced listeners require stronger cortical engagement (Reiterer et al., 2011; Zhang et al., 2005). However, that work could not isolate neural signatures at particular linguistic stages. Recent studies have successfully isolated neural signatures of various linguistic levels based on speech-EEG synchrony (cortical tracking; Obleser and Kayser, 2019) from a single electrophysiological recording. Such measures were derived based on low-frequency cortical responses to natural speech from audiobooks (Brodbeck et al., 2018a; Di Liberto et al., 2015; Khalighinejad et al., 2017) and cartoons (Di Liberto et al., 2018b; Jessen et al., 2019), which were recorded non-invasively from both children and adults. Here, we adopted the same framework to investigate how proficiency shapes hierarchical cortical encoding in L2 subjects and how hierarchical encoding differs from L1 subjects. Our analysis focused on speech processing at the levels of sound acoustics (Ahissar et al., 2001; Lalor and Foxe, 2010), phonemes (Di Liberto et al., 2015; Khalighinejad et al., 2017), phonotactics (statistics on phoneme sequences Brodbeck et al., 2018a; Di Liberto et al., 2019), and semantics (Broderick et al., 2018; Hagoort and Brown, 2000; Kutas and Federmeier, 2011). We hypothesized that the neural encoding of all three levels of linguistic properties would be modulated by L2 proficiency, becoming more native-like without fully converging (Han, 2004; Selinker, 1972). A different progression of this learning effect was expected for distinct linguistic levels. Specifically, we predicted that phoneme and phonotactic responses, which benefit from but do not require sentence comprehension, would show a continuous progression starting from the earliest stages of learning, partly as a form of implicit learning (Conway et al., 2010). Furthermore, even when two spoken languages are very different, there is some level of phonological and phonotactic overlap. As such, we predicted that phoneme and phonotactic responses would emerge even with listeners who do not understand English due to the encoding of the same or similar information in their native language. Semantic-level encoding is different in this regard. Specifically, whereas semantic encoding was hypothesized to increase with proficiency (McLaughlin et al., 2004), we predicted a most prominent change from no encoding to strong encoding at an intermediate level of proficiency as the comprehension of a few words facilitates the understanding of neighboring words (e.g., semantic priming; Kellenbach et al., 2000; Osterhout and Holcomb, 1995); thus, constituting a turning point beyond which comprehension increases drastically.

To shed light on the neural mechanisms underlying the encoding of linguistic features, the present study combines objective neural indices of acoustic and linguistic processing to assess the differences between L2 subjects with varying proficiency levels during a task requiring the individual to listen to natural speech. We expected hierarchical linguistic encoding in L2 participants to change with proficiency. While the present study primarily investigates the effect of proficiency within the L2 group, we also evaluated our results in the context of the previous literature on L1 listeners (Brodbeck et al., 2018a; Broderick et al., 2018; Di Liberto et al., 2015; Khalighinejad et al., 2017); this evaluation was conducted with respect to the hypothesis that neural linguistic encoding is more similar to that of L1 listeners in nonnative listeners with higher proficiency than to that of those with lower proficiency.

## 2. Materials and methods

### 2.1. Participants

Fifty-one healthy subjects (twenty-four were male, all were aged between 18 and 60 years, median age = 24 and mean age = 27.5; forty-eight were right-handed) who learned English as a second language (or that did not speak English) and were native Standard Chinese (Mandarin) speakers participated in the EEG experiment (*L2 group*). L2 participants were asked to take a standardized 20-minute test of receptive skills in American English before the experiment. According to the results of this assessment, each participant was assigned to one of six proficiency groups according to the Common European Framework of Reference for Languages framework (CEFR): A1, A2, B1, B2, C1, C2 (from low to high proficiency). A, B, and C levels indicate *basic, independent*, and *proficient* users, respectively. The A1 group included participants with very little or no English understanding. The recruitment of participants continued until 17 participants were identified for each A, B, and C group (Supplementary Figure 1B). Two subjects were excluded because of issues with their EEG recordings (data could not be synchronized because of missing trigger signals).

We also analyzed EEG data from twenty-two native English speakers (twelve were male, all were between 18 and 45 years, twenty were right-handed; *L1 group*), originally collected for a previous study with the same experimental setup and location (Khalighinejad et al., 2017), who listened to the same continuous English speech stimuli presented to the L2 group in the present experiment. All subjects (in both L2 and L1 groups) reported having normal hearing and had no history of neurological disorders. All subjects provided written informed consent and were paid for their participation. The Institutional Review Board of Columbia University at Morningside Campus approved all procedures.

### 2.2. Stimuli and behavioral tasks

EEG data were collected in a sound-proof, electrically shielded booth in dim light conditions. Participants listened to short stories from a children’s story book (Hank the Cowdog), narrated in English by two native speakers (1 male) while minimizing motor movements and maintaining visual fixation on a crosshair at the center of the screen. All participants listened to identical auditory stimuli. The stories continued naturally between trials, with an alternation between the male and the female narrators to minimize speaker-specific effects. Stimuli were presented at a sampling rate of 44,100 Hz, monophonically, and at a fixed comfortable volume from one loudspeaker in front of the participant. Each session consisted of 20 experimental blocks (3 minutes each), and short breaks were planned every 4 blocks. Participants were asked to focus their attention on speech material from seven audio-stories that were presented in a random order. Engagement with the speech material was assessed by means of behavioral tasks. During speech listening, L2 participants were asked to perform a phrase-repetition detection task. The detection of repeated sounds has been successfully used in many studies using nonspeech and nonsense speech sounds. Here, the last two to four words were repeated immediately after the end of some of the sentences (1–5 per block). Given that our target was monitoring attention, a finger-tip clicker was used to count the repetitions so that they would be engaged in detection not counting, which would instead require additional memory resources and, potentially, reduce their engagement in the main listening task. Furthermore, L2 participants were asked three questions at the end of each block (Supplementary Fig. 1). *First*, participants were shown a list of eight words and asked to identify, among those, 3–5 words that occurred with high frequency in the speech sentence. *Second*, we asked whether the last sentence of the block was spoken by a male or female speaker. *Third*, participants were asked to report the count associated with the phrase-repetition detection task at the end of each block. EEG data corresponding to the phrase repetition were excluded from the analysis to remove contamination due to motor action. To assess attention in L1 participants, three questions about the content of the story were asked after each block. All L1 participants were attentive and could all answer at least 60% of the questions correctly.

### 2.3. EEG recordings and preprocessing

EEG recordings were performed using a g.HIamp biosignal amplifier (Guger Technologies) with 62 active electrodes mounted on an elastic cap (10–20 enhanced montage). EEG signals were recorded at a sampling rate of 2 kHz. An external frontal electrode (AFz) was used as the ground, and the average of two earlobe electrodes was used as a reference. EEG data were filtered online using a high-pass Butterworth filter with a 0.01 Hz cutoff frequency to remove DC drift. Channel impedances were kept below 20 kΩ throughout the recording.

Neural data were analyzed offline using MATLAB software (Math-Works Inc.). EEG signals were digitally filtered between 1 and 15 Hz using a Butterworth zero-phase filter (order 2 + 2 and implemented with the function *filtfilt*), and downsampled to 50 Hz (similar to relevant previous work, e.g., Broderick et al., 2018; Di Liberto et al., 2015). EEG channels with a variance exceeding three times that of the surrounding channels were replaced by an estimate calculated using spherical spline interpolation.

### 2.4. Speech features

In the present study, we measured the coupling between EEG data and various properties of speech stimuli. These properties were extracted from the stimulus data based on previous research. First, we defined a set of descriptors summarizing *low-level acoustic properties* of the music stimuli. Specifically, a time-frequency representation of the speech sounds was calculated using a model of the peripheral auditory system (Chi et al., 2005) consisting of three stages: (1) a cochlear filter bank with 128 asymmetric filters equally spaced on a logarithmic axis, (2) a hair cell stage consisting of a low-pass filter and a non-linear compression function, and (3) a lateral inhibitory network consisting of a first-order derivative along the spectral axis. Finally, the envelope was estimated for each frequency band, resulting in a two-dimensional representation simulating the pattern of activity on the auditory nerve (Wang and Shamma, 1994) (the relevant MATLAB code is available at https://isr.umd.edu/Labs/NSL/Software.htm). This *acoustic spectrogram* (**S**) was then resampled to 16 bands (Di Liberto et al., 2015; Lesenfants et al., 2019). A *broadband envelope* descriptor (**E**) was also obtained by averaging all envelopes across the frequency dimension. Finally, the *halfway rectified first derivative of the broadband envelope* (**E’**) was used as an additional descriptor, which was shown to contribute to speech-EEG mapping and was used here to regress out the most acoustic-related responses as much as possible (Daube et al., 2019).

Additional speech descriptors were defined to capture neural signatures of higher-order speech processing. The speech material was segmented into time-aligned sequences of phonemes using the Penn Phonetics Lab Forced Aligner Toolkit (Yuan and Liberman, 2008). Then, the phoneme alignments were manually corrected using Praat software (Boersma and Weenink, 2009). *Phoneme onset* times were then encoded in an appropriate univariate descriptor (**Pon**), in which ones indicated onsets and all other time samples were labeled with zeros. An additional descriptor was also defined to distinguish between *vowels and consonants* (**Pvc**). Specifically, this regressor consisted of two vectors, similar to **Pon**, but labeling either vowels or consonants only. While **Pon** was shown to improve speech-neural mapping compared to when only acoustic features were used to describe the stimulus (Brodbeck et al., 2018a, but see Daube et al., 2019), additional information on phoneme categories remained that contributed to that mapping (Di Liberto et al., 2015; Mesgarani et al., 2014). This information was encoded in a 19-dimensional descriptor indicating the *phonetic articulatory features* corresponding to each phoneme (**Phn**). Features indicated whether a phoneme was voiced, unvoiced, sonorant, syllabic, consonantal, approximant, plosive, strident, labial, coronal, anterior, dorsal, nasal, fricative, obstruent, front (vowel), back, high, or low (Chomsky and Halle, 1968; Ladefoged and Johnson, 2014; Supplementary Table 2). The **Phn** descriptor encoded this categorical information as step functions, with steps corresponding to the starting and ending time points for each phoneme. Note that a step function includes information on the duration of a phoneme, thus including information about its identity, since certain phonemes are longer than others on average. For this reason, we purposely did not include that information in **Pon**, which describes only the timing of the phoneme onsets and not the phoneme categories.

Next, we encoded *phonotactic probability* information in an appropriate two-dimensional vector (**Pt**) (Brodbeck et al., 2018a; Di Liberto et al., 2019). In a given language, certain phoneme sequences are more likely to be valid speech tokens than others. The likelihood of a phoneme sequence p1...n being a valid speech token can be estimated with statistical models based on language-specific rules. Here, probabilities were derived by means of the BLICK computational model (Hayes and Wilson, 2008), which estimates the probability of a phoneme sequence belonging to the English language. This model is based on a combination of explicit theoretical rules from traditional phonology and a MaxEnt grammar (Goldwater and Johnson, 2003), which find optimal weights for such constraints to best match the phonotactic intuition of native speakers. The phonotactic probability was derived for all phoneme sub-sequences within a word (ph_1_.. *_k_*, 1 ≤ *k* ≤ *n*, where *n* is the word length) and used to modulate the magnitude of a phoneme onset vector (**Pt_1_**). The calculation proceeds in several steps (Hayes and Wilson, 2008). First, the score (*h*) of a phonological representation *x* = *ph*_1*..k*_ is calculated as $h\left(x\right)=\sum_{i=1}^{m}w_{i}*C_{i}\left(x\right),$ where *m* is the number of phonotactic constraints, *w_i_* is the weight of the i^th^ constraint, and *C_i_*(*x*) is the number of times *x* violates that constraint. Second, the MaxEnt value (*P*^∗^) is calculated as P*(*x*) = exp(−*h*(*x*)). Finally, the probability of *x* is calculated as $P\left(x\right)=P*\left(x\right)/\sum_{y\in \Omega }P*\left(y\right)$, where Ω represents all possible values of x. A second vector was produced to encode the change in phonotactic probability due to the addition of a phoneme (ph_1...*k*_ - ph_1...*k-1*_, 2 ≤ *k* ≤ *n*) (**Pt_2_**).

Finally, a semantic dissimilarity descriptor was calculated for content words using word2vec (Baroni et al., 2014; Mikolov et al., 2013), a state-of-the-art algorithm consisting of a neural network for the prediction of a word given the surrounding context. In this specific application, a sliding window of 11 words was used; in this window, the central word was the output, and the surrounding 10 words were the input. This approach is based on the “distributional hypothesis” that words with similar meanings occur in similar contexts; this method uses an artificial neural network approach to capture this phenomenon. This network has a 400-dimension hidden layer that is fully connected to both input and output. For our purposes, the weights of this layer were the features used to describe each word in this 400-dimensional space, thus capturing the co-occurrence of a content word with all others. In this space, words that share similar meanings have a closer proximity. The semantic dissimilarity indices were calculated by subtracting the Pearson’s correlation between a feature vector for a word and the average feature vector across all previous words in that particular sentence from 1 (the first word in a sentence was instead correlated with the average feature vector for all words in the previous sentence). Thus, if a word is unlikely to co-occur with the other words in the sentence, it should not correlate with the context, resulting in a higher semantic dissimilarity value. The semantic dissimilarity vector (**Sem**) marks the onset of content words with their semantic dissimilarity index.

### 2.5. Computational model and data analysis

**Fig. 1.**
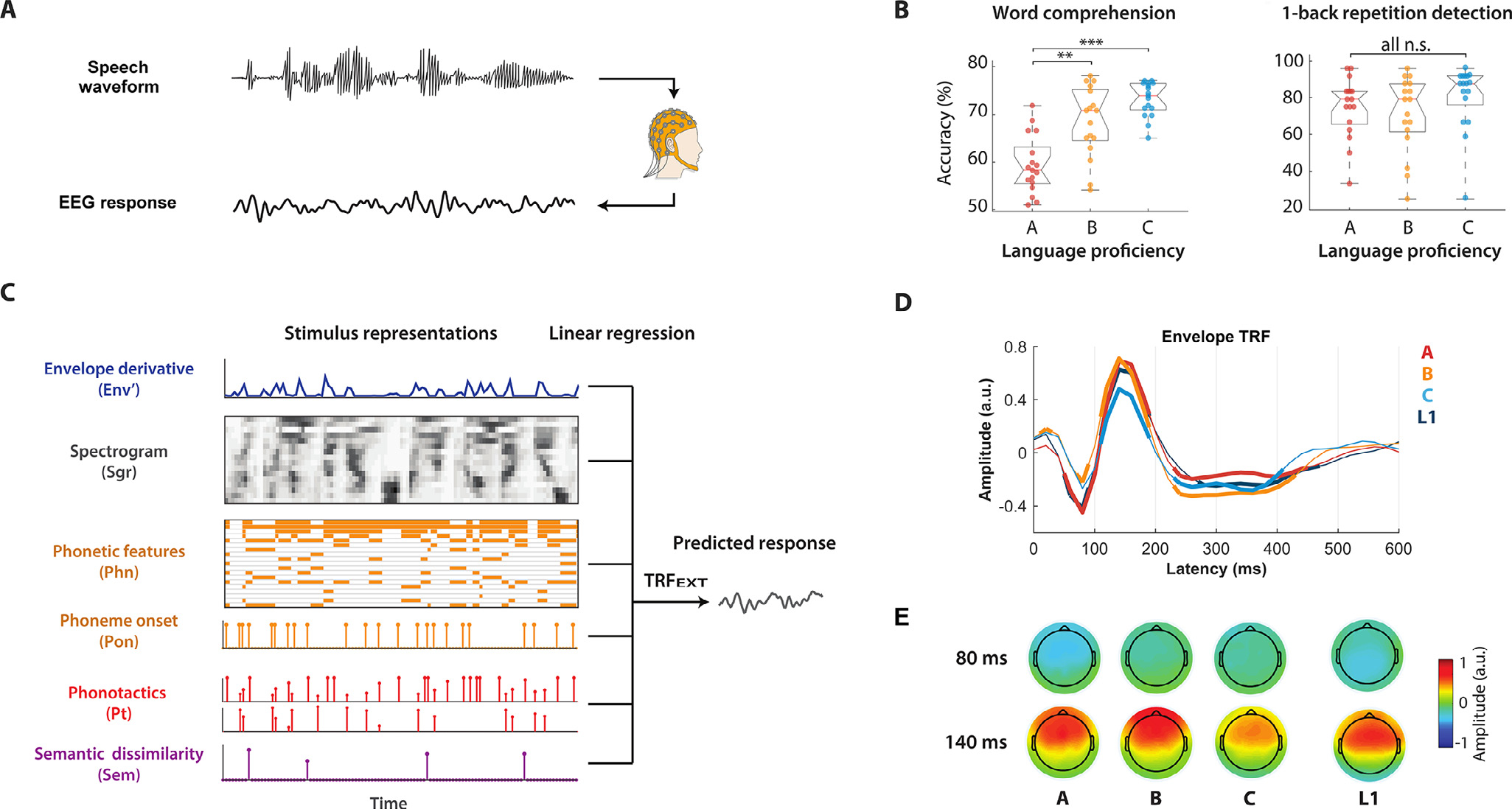
Investigating the hierarchical cortical encoding of language with the temporal response function (TRF) analysis framework. (A) Multichannel EEG signals were recorded as participants listened to audio stories. Participants were asked to press a clicker counter when they detected a one-back phrase repetition (2–4 words), which occurred 1–5 times per experimental block. At the end of each block, participants were asked to report the number of repetitions, to identify words that were spoken during the block from a list of eight and to indicate the gender of the speaker. (B) Results for the word comprehension and the 1-back detection task. Significant group differences (ANOVA, ** p<0.01, *** p<0.001) were measured for the ‘word comprehension’ score that positively correlated with proficiency. No significant effects emerged for ‘one-back tasks’ and ‘gender identification’ as they were independent from the proficiency levels. The gender identification result was not reported, as accuracy was larger than 95% for all participants. (C) Acoustic and linguistic information were extracted from the stimulus and encapsulated into data vectors and matrices. Multivariate linear regression was used to identify a linear fit that optimally predicted the EEG signal from features at multiple linguistic levels (EXT). The same procedure was also run on a more compact set of descriptors (ALL), which differed in that Sgr and Phn were replaced by Env (broadband envelope instead of a 16-band spectrogram) and Pvc (indicator variables for only vowels and consonants rather than for a 19-dimensional set of phonetic features), respectively. (D) Envelope TRF weights (TRF _Env_) averaged across all EEG channels at peristimulus time latencies from 0 to 600 ms. TRF _Env_ was part of a model that was fit by including features at all other levels of interest (ALL). Thick lines indicate weights that were significantly different from zero across all subjects of a group (p < 0.05, Wilcoxon signed rank test, FDR corrected). Significant effects of group emerged on the peak-to-peak amplitude of the N1-P2 TRF complex (one-way ANOVA, p = 0.03). No significant effect of group emerged at any individual time latency (point-by-point one-way ANOVA, p > 0.05). (E) Topographies of the TRF weights across channels for two selected time latencies.

**Fig. 2.**
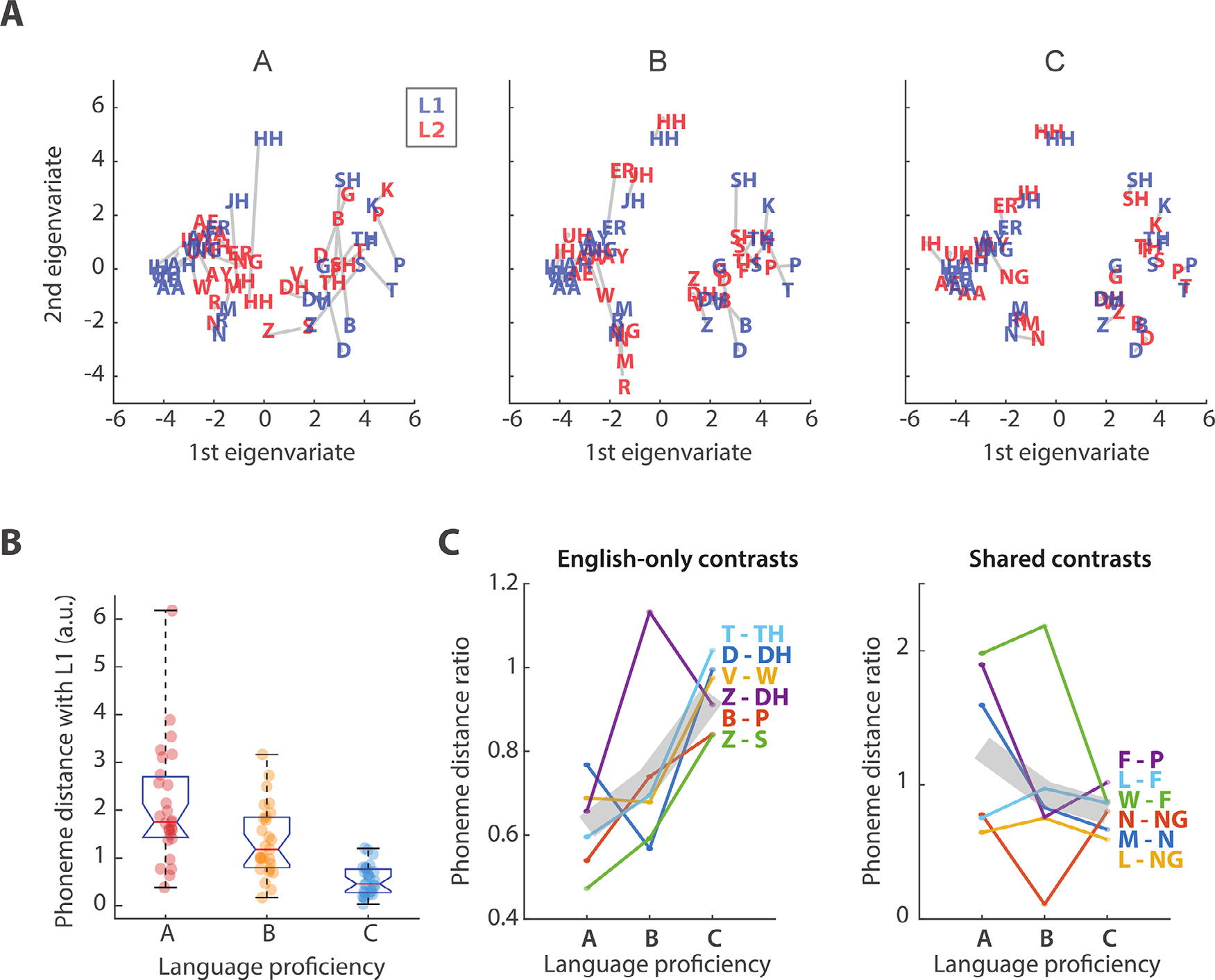
Effect of proficiency on L2 phoneme encoding. (A) Phoneme distance maps (PDMs) based on the TRF _Ph_ weights at electrode Cz and peristimulus time latencies from 0 to 600 ms. Blue and red colors indicate phonemes for L1 and L2 participants, respectively. (B) Distance between L1 and L2 phonemes for each language proficiency group. A significant effect of proficiency was measured on the L1-L2 phoneme distance (one-way ANOVA, F(1.4, 54.1) = 22.8, p = 1.6*10^−8^). Error bars indicate the SE of the mean across phonemes. (C) Distance between phoneme pairs for each proficiency level. The left panel shows results for contrasts existing in English but not in Standard Chinese; in these contrasts, we expected increasing discriminability with proficiency due to learning. The right panel shows distances for contrasts that exist both in English and Standard Chinese; in these contrasts, we did not expect a learning effect. Values were divided by the distance for L1 participants. Gray lines indicate the mean across all selected phonemic contrasts. (For interpretation of the references to color in the text, the reader is referred to the web version of this article.)

A single input event at time *t_0_* affects the neural signals for a certain time window [*t_1_, t_1_* + *t*_win_], with *t 1* ≥ 0 and *t win >* 0. Temporal response functions (TRFs) were fit to describe the speech-EEG mapping within that latency window for each EEG channel (TRF; Ding et al., 2014; Lalor et al., 2009). We did this by means of a regularized linear regression (Crosse et al., 2016) that estimates a filter that allows us to optimally predict the neural response from the stimulus features (forward model; Fig. 1 C). The input of the regression also included time-shifted versions of the stimulus features so that the various time lags in the latency window of interest were all simultaneously considered. Therefore, the regression weights reflect the relative importance between time latencies in the stimulus-EEG mapping and were studied here to infer the temporal dynamics of the speech responses (see Figs. 1 and 2). Here, a time-lag window of 0–600 ms was used to fit the TRF models, which was considered to contain most of the EEG responses to the speech of interest. Specifically, neural processing of the acoustic and linguistic features of interest was expected to emerge after the speech sound was presented (*t _1_ >* 0), whereas the component of interest with the longest latency, the semantic dissimilarity TRF, was expected to be mostly complete by a latency of 600 ms (Broderick et al., 2018). The reliability of the TRF models was assessed using a leave-one-out cross-validation procedure (across trials), which quantified the EEG prediction correlation (Pearson’s *r*) on unseen data while controlling for overfitting. The TRF model calculation included a Tikhonov regularization, which involves the tuning of a regularization parameter (*λ*) that was conducted by means of an exhaustive search of a logarithmic parameter space from 0.01 to 10 ^10^ on the training fold of each cross-validation iteration (Crosse et al., 2016). Note that the correlation values were calculated with the noisy EEG signal; therefore, the *r* -scores could be highly significant even though they have low absolute values (*r* ~ 0.1 for sensor-space low-frequency EEG; Daube et al., 2019; Di Liberto et al., 2015; Lesenfants et al., 2019).

Stimulus descriptors at the levels of acoustics, phonemes, phonotactics, and semantics were combined in a single TRF model fit procedure. This strategy was adopted with the goal of discerning EEG responses at different processing stages. For example, a TRF derived with **Pt** alone could reflect EEG responses to both phonotactics and the acoustic envelope **Env**, as the two vectors are correlated. A TRF based on the combination of **Pt** and **Env** would instead discern their respective EEG contributions; hence, the weights for **Pt** will reflect responses that are most relevant to phonotactics, whereas the responses that are more related to **Env** than to **Pt** will become less prominent in TRF_Pt_. Though this separation is straightforward for independent features, it is certainly not straightforward for this experiment. In fact, this operation is expected to strengthen the representation of the neural encoding of features of interest and not to achieve perfect separation; perfect separation could be attained when working with independent features, which is certainly not the case here.

Here, individual-subject TRFs were fitted by combining **Env, Env’, Pvc, Pon, Pt**, and **Sem** (stimulus descriptor **ALL**). We also fit TRF models with an extended stimulus descriptor (**EXT**) including **Sgr, Env’, Phn, Pon, Pt**, and **Sem**, which provided us with a higher level of detail on spectrotemporal and phonological speech features at the cost of higher dimensionality (see Supplementary Figs. 2 **and** 3). The combined stimulus descriptor had 40 dimensions, which had to be multiplied by the number of time lags (30 when the sampling frequency was 50 Hz) to obtain the dimensionality of the TRF input. For this reason, we conducted all analyses on the reduced stimulus set **ALL**, whereas the **EXT** descriptor was used to assess spectrotemporal and phoneme TRFs. As data collection was conducted so that the three proficiency groups A, B, and C were balanced and sufficiently large to derive the TRFs of interest, all TRF analyses were conducted according to this 3-way grouping of the L2 participants. The subsequent decoding analysis was conducted instead by taking into consideration the more detailed 6-way grouping (A1, A2, B1, B2, C1, and C2), as regression decoding (see the *Proficiency-level decoding* subsection) focused on individual-subject decoding rather than between-group analyses;, furthermore, it tolerates small imbalances across groups.

The TRF weights constitute good features to study the spatiotemporal relationship between a stimulus feature and the neural signal. The TRF weights represent the impulse response of the estimated linear system, and as such, their values at a given latency *t_1_* correspond to a positive or negative deflection of the EEG electrical signal *t_1_* ms after hearing an impulse sound at time zero. As such, TRFs can be interpreted similarly to ERPs, as they are both defined in a channel x latency domain, with the advantage that they can describe the EEG response to a continuous stimulus. For ERPs, prior knowledge and hypotheses can be used to restrict the channel and latency space to investigate, thus simplifying the analysis. Here, the TRF analyses for Env, Pt, and Sem were especially focused on five midline electrodes [Oz, Pz, Cz, Fz, and Fpz] and to the latency window [0, 600] ms. The five electrodes were chosen based on prior studies indicating that the TRFs of interest would emerge as components centered on the vertical midline, whereas the latency window was chosen as it was sufficient to capture the components of interest for Env, Phn, Pt, and Sem (Brodbeck et al., 2018a; Broderick et al., 2018; Di Liberto et al., 2019, 2015).

Another difference between TRFs and ERPs is that TRFs can be multivariate, thus describing the impulse responses for different contributors to the EEG signal. The study of a multivariate speech descriptor, such as **Phn**, could benefit from the derivation of indices that summarize the impact of those multiple dimensions into single values. One solution was to use the EEG prediction correlation values to quantify the goodness of fit for a multivariate TRF model. Here, we considered the relative enhancement in EEG prediction correlation when **Phn** was included in the model (using the **ALL** feature-set), thus allowing us to discern the relative contribution of phonetic features to the neural signal. This isolated index of phoneme-level processing was previously shown to correlate with psychometric measures of phonological skills (Di Liberto et al., 2018b).

Further analyses were conducted by using a generic modeling approach (Di Liberto and Lalor, 2017). Specifically, one generic TRF model was derived for groups A, B, C, and L1 by averaging the regression weights from all subjects within the group. Then, EEG data from each left-out subject (whose data were not included in the generic models) were predicted with the four models. The four prediction correlations were used as indicators of how similar the EEG signal from a subject was to the one expected for each of the four groups, providing us with a simple classifier.

### 2.6. Phoneme distance maps (PDMs)

We sought to study the effect of proficiency on phonological perception by projecting TRF_Phn_ onto a space in which distance represents the perceptual discriminability between pairs of phonemes. The regression weights for TRF_Phn_ were represented in a 19-dimensional space of phonetic features. Each phoneme could be described as a particular linear combination of phonetic features. This allowed for the derivation of a linear transformation matrix describing the linear mapping from phonetic features to phonemes, which we used to rotate TRF_Phn_ to the phoneme domain (Supplementary Fig. 3). Then, a classical multidimensional scaling (MDS) was used to project the TRF_Phn_ (phonemes were considered as objects and time latencies were considered as dimensions) onto a multidimensional space for each proficiency group, in which distances represented the discriminability of particular phonetic contrasts in the EEG signal. The result for each L2 proficiency group was then mapped to the average L1-MDS space by means of a Procrustes analysis (MATLAB function *procrustes)*. This analysis allowed us to project the L2 phoneme maps for different proficiency levels to a common multidimensional space where they could be compared quantitatively; we call these maps phoneme distance maps (*PDMs)*.

### 2.7. Proficiency-level decoding

Support vector regression (SVR) with a radial basis function kernel was used to decode the proficiency level of L2 participants. The output of the regression was the proficiency level, a continuous variable. As such, the decoding output was a decimal number, even though proficiency levels in the training data could have only six possible values corresponding to A1, A2, B1, B2, C1, and C2. The input of the SVR was the concatenation of 26 features derived from the TRF analysis described in the previous section. All features were continuous variables. The neural encoding of acoustic and linguistic features could be assessed based on the model weights or on the EEG prediction correlation values (Crosse et al., 2016), and the latter could be calculated with subject-specific models or generic models (averaged across multiple participants; Di Liberto and Lalor, 2017). Here, the 26 features were selected according to previous studies with L1 participants (Brodbeck et al., 2018a; Broderick et al., 2018; Di Liberto et al., 2019, 2015) as they capture the main TRF components in the model weights (9 features), subject-specific EEG prediction correlations (5 features), and generic models EEG prediction correlations (12 features; see also Di Liberto and Lalor, 2017).

Each feature had multiple dimensions, such as EEG electrodes and time latencies. A multilinear principal component analysis (MPCA) was performed to summarize each of them with a single vector. Specifically, given the TRF weights for all electrodes and latencies for a particular feature (e.g., envelope), MPCA was performed, and the first component was retained (see Supplementary Table 1 for details on the explained variance for each feature). This operation was performed for the TRFs corresponding to envelope, phoneme onsets, phonetic features, phonotactics, and semantic dissimilarities, resulting in five distinct vectors. Based on previous TRF studies and our initial hypotheses, we complemented the result of this lossy compression by adding distinctive features that summarized specific aspects of interest of the TRFs. For speech acoustics, we included information on the power spectrum of the TRF (the EEG responsiveness to 16 logarithmically spaced sound frequencies) by collapsing the weights in TRF_ALL_ corresponding to **Sgr** values across the time-latency dimension. MPCA was then conducted on the resulting values to quantify this spectral feature with a single value per subject. For phonotactics and semantic dissimilarity, the strength of the main TRF components was summarized by averaging the regression weights over selected time windows and electrodes where they were strongest in the current data (80–140 and 300–700 for **Pt** and 300–700 for **Sem** at Fz, Cz, and Oz, respectively).

Additional features were based on the EEG prediction correlations calculated by training and testing TRF models on each participant separately (with leave-one-out cross-validation across recording blocks; see previous section). This procedure provided us with a correlation score for each electrode, which was then summarized with a single value by performing MPCA and retaining the first component. This procedure provided us with four features for EEG predictions based on **Env, Phn, Pt, Sem**. Note that, different from the TRF weight features, these EEG prediction correlations were calculated for each feature separately. A fifth feature was derived by measuring the increase in EEG prediction correlations when **Phn** was included or was not in the stimulus set together with **Env’** and **Sgr** (**PhnEnv’Sgr-Env’Sgr**). This subtraction was considered to constitute an isolated measure of phoneme-level processing (Di Liberto et al., 2018b, 2015). The decoding result did not change when using **PhnEnv’Sgr-Phn_Shu_ Env’Sgr** instead, where **Phn_Shu_** is a random phoneme vector. Finally, EEG signals from a subject were also predicted with TRF models fit on all other subjects, grouped in **A, B, C**, and **L1**, with the rationale that the EEG data from a given subject should be best predicted by TRF models from subjects of the same group. This approach, which has been referred to as average modeling or generic modeling (Di Liberto and Lalor, 2017; Jessen et al., 2019), provided each subject with a score for each group and for each feature of interest. Here, we selected **Env, PhnEnv’Sgr-Env’Sgr**, and **Sem**. MPCA was then used for dimensionality reduction for the other features, providing us with twelve features (4 groups and 3 predicting features).

SVR was used to decode the L2 proficiency level, for the binary classification L1 versus L2 data, or for the binary classification L1 versus C-level L2 data with leave-one-out cross-validation. A backward elimination procedure was used to identify the optimal set of features that minimize the mean squared error (MSE) of the decoded proficiency levels. Specifically, starting from a set containing all the features, the regressor whose exclusion produced the larger decrease in MSE was removed at each step. This procedure continued as long as there was at least 5% improvement in the MSE score (please see Supplementary Table 1 for a full list of features and information on the selected feature for the L2 decoding and on L1 vs. L2 classification procedures).

### 2.8. Statistical analysis

Statistical analyses were performed using Wilcoxon tests for pairwise comparisons. Correction for multiple comparisons was applied where necessary via the false discovery rate (FDR) approach. Three-way ANOVA was used to assess the effect of proficiency on the TRF weights with latency and electrode as additional fixed factors. One-way ANOVA was used when testing the significance of an effect over multiple (> 2) groups when comparisons did not involve multiple latencies and electrodes. The values reported use the convention *F* (*df, df_error_*). Greenhouse-Geisser correction was applied when the assumption of sphericity was not met (as indicated by a significant Mauchly’s test result). FDR-corrected Wilcoxon tests were used after ANOVA for post hoc comparisons.

## 3. Results

EEG signals were recorded as participants listened to audio stories. The experiment involved behavioral tasks to monitor engagement with the speech material (Fig. 1 B and Supplementary Fig. 1). As expected, the word comprehension score increased significantly with proficiency (ANOVA, *F* (1.8, 44.3) = 24.1; *p* = 5.4^∗^10^−8^, post hoc comparisons: *p* A-vs-B = 0.003, *p* B-vs-C = 0.084, *p* A-vs-C < 0.001). However, all subjects were able to perform the gender identification and one-back tasks with similar accuracy across proficiency levels, suggesting a comparable degree of engagement among participants across groups (*gender identification task*: ANOVA, *F* (1.4, 34.7) = 0.1, *p* = 0.90; *one-back task*: ANOVA, *F* (1.9, 45.9) = 1.1; *p* = 0.34).

### 3.1. Hierarchical cortical encoding of nonnative speech

To investigate the low-versus higher-level brain processing of speech, we fit forward TRF models to assess the coupling between the low-frequency cortical signals (1–15 Hz) and progressively more abstract properties of the linguistic input. The combination of multiple speech features in a single multivariate model allowed us, for the first time, to assess the hierarchical processing of L2 speech from a single EEG recording session based on natural speech. The stimulus descriptor included Env, Env’, Pon, Pvc, Pt, and Sem (ALL; see Methods). This combination of features allowed us to capture and discern EEG variance corresponding to various hierarchical stages while using a low-dimensional descriptor (8 dimensions). We also fit TRF models with an extended stimulus descriptor (EXT) that included Sgr, Env’, Pon, Phn, Pt, and Sem, which provided us with a higher level of detail in the spectrotemporal and phonological processing of speech. However, this increased dimensionality of the model (40 dimensions) makes fitting the model more challenging. Leave-one-out cross-validation indicated that the resulting TRF models could reliably predict the EEG signal for all subjects (*r ALL >* r ALL_SHUFFLE and *r EXT >* r EXT_SHUFFLE, *p* < 0.01, permutation test in which input sentences were randomly shuffled, N = 100; EEG prediction correlations were averaged across all electrodes).

Model weights corresponding to the Env descriptor (TRF_Env_, a subset of TRF_ALL_) showed *significant effects of proficiency* (three-way ANOVA with latency, electrodes, and proficiency as factors, *F* (2,6670) = 8.65, *p* = 1.7*10 ^−4^), latency (*F* (28,6670) = 90.22, *p* < 10 ^−100^) and electrode (*F* (4,6670) = 3.25, *p* < 0.011) with a significant proficiency*latency interaction (*F* (56,6670) = 4.61, *p* = 4.1*10^−27^) and latency*electrode interaction (*F* (112,6670) = 10.60, *p* < 10^−100^) but no significant proficiency*electrode interaction (*F* (8,6670) = 0.69, *p* = 0.71). Fig. 1 D, E shows the model weights corresponding to the Env descriptor (part of TRF _ALL_) after averaging across all electrodes and all subjects within each proficiency group (A, B, C, and L1). TRFs for the four groups appeared temporally synchronized, which was expected for cortical responses to low-level acoustics. While proficiency effects emerged on the TRF_Env_ magnitude did not survive correction for multiple comparisons at individual time-latencies (point-by-point one-way ANOVA with FDR correction), significant effects of group emerged on the peak-to-peak amplitude of the N1-P2 TRF complex when considering the average TRF across all electrodes (one-way ANOVA, *p* = 0.03) as well as on individual electrodes (one-way ANOVA with FDR correction, *p* < 0.05 on 11/64 electrodes). We also tested whether the envelope response in L2 participants became more similar to that of native speakers with proficiency. To do so, we measured the Pearson’s correlation scores between the TRF_Env_ for each L2 subject and the average TRF _Env_ weights across all L1 participants. This measure of similarity between L1 and L2 subjects did not show any significant difference between the A, B, and C groups (*p >* 0.05).

Although envelope TRFs have proven to be robust and have contributed to the study of various aspects of auditory perception (Bednar and Lalor, 2020; Crosse et al., 2015; Hausfeld et al., 2018; O’Sullivan et al., 2014), we also modeled the low-level auditory responses by considering the acoustic spectrogram (Sgr), which was shown to be a better predictor of the EEG signal (Daube et al., 2019; Di Liberto et al., 2015). However, observing TRF_Sgr_ (part of TRF_EXT_) for different auditory frequency bands did not lead to new clear-cut insights in this case; thus, the rest of the manuscript focuses on the envelope TRF results.

### 3.2. Effect of proficiency on the cortical encoding of phonemes in L2 listeners

Phonetic feature information was represented by the categorical descriptor **Phn**, which marked the occurrence of a phoneme with a rectangular pulse for each corresponding phonetic feature (see Methods) (Di Liberto et al., 2015). TRFs were fit for each subject by combining the **Phn** descriptor with all others in the **EXT** feature set. The weights corresponding to the descriptor of interest, TRF_Phn_, were extracted from TRF _EXT_. In this case, the other descriptors served as nuisance regressors, meaning that they reduced the impact of acoustic-, phonotactic- and semantic-level responses on TRF_Phn_. The effect of proficiency was assessed in L2 participants by measuring the change in TRF _Phn_ between proficiency levels A, B, and C. PDMs were derived based on an MDS analysis that projected the TRF _Phn_ weights onto a multidimensional space, with distances representing the discriminability of particular phonetic contrasts in the EEG signal (Fig. 2 A; see Methods – Phoneme distance maps). The effect of proficiency on the PDMs was studied by measuring the distance between L1 and L2 maps (Fig. 2 B) and by measuring the change in the pairwise phoneme distance within L2 maps (Fig. 2 C and Supplementary Fig. 4). Fig. 2 B shows the average L1-L2 distance across all phonemes for each L2 participant, with blue and red fonts indicating phonemes for L1 and L2 participants, respectively. Shorter L1-L2 distances were measured in the PDMs for increasing L2 proficiency levels (Fig. 2 B: ANOVA, *F* (1.4, 54.1) = 22.8; *p* = 1.6*10^−8^), indicating an effect of proficiency on the TRF_Phn_, with a progressive convergence of the PDM to that for native listeners.

Unknown phonemes have been shown to be perceived by L2 speakers as the closest existing phonemic neighbor in their L1, thus presenting challenges in discrimination (Flege, 1988; Guion et al., 2000). To test whether phonetic contrasts that do not exist in Standard Chinese (the native language of L2 subjects) became more discriminable in the EEG data with increasing proficiency, we measured the distance between pairs of phonemes in PDM space for each proficiency group. As TRF_ph_ was previously shown to be particularly sensitive to contrasts between consonants but not between vowels (Di Liberto et al., 2018a, 2015), phonemic discriminability was assessed for all pairs of consonants in the English phonemic inventory. We expected discriminability to increase with proficiency when considering phonemic contrasts that exist in English but not in Standard Chinese, thus reflecting the improved discrimination skills of L2 listeners. Our data were sensitive to this learning process, as we measured significant effects of L2 proficiency on the discriminability of phoneme pairs that do not exist in Standard Chinese (ANOVA: *F* (1.6, 150.3) = 3.8, *p* = 0.024; Supplementary Fig. 4) and for pairs in which only one phoneme exists in Chinese (ANOVA: *F* (1.4, 245.8) = 4.4, *p* = 0.013), whereas no significant effects were measured for contrasts that exist in Chinese and, as such, L2 participants were sensitive to individuals without any knowledge of the English language (ANOVA *F* (1.26, 90.9) = 0.9, *p* = 0.39). Figs. 2 C shows this result for selected phonetic contrasts that are of particular importance for correct English comprehension as they occur in minimal pairs (words differentiated by only one phoneme, e.g., “bat ” /bæt/, “pat ” /pæt/). The Fig. shows six selected English-only contrasts (T vs. TH, D vs. DH, V vs. W, Z vs. DH, B vs. P, and Z vs. S), all of which exhibit increased discriminability when comparing the A and C proficiency-level groups, and six selected phonemic contrasts belonging to both English and Standard Chinese languages (F vs. P, L vs. F, W vs. F, N vs. NG, M vs. N, and L vs. NG), which did not show any consistent change with proficiency. In this case, distance values for each phoneme pair were normalized based on the L1 map for visualization.

### 3.3. Proficiency modulates phonotactic responses at both short and long latencies

**Fig. 3.**
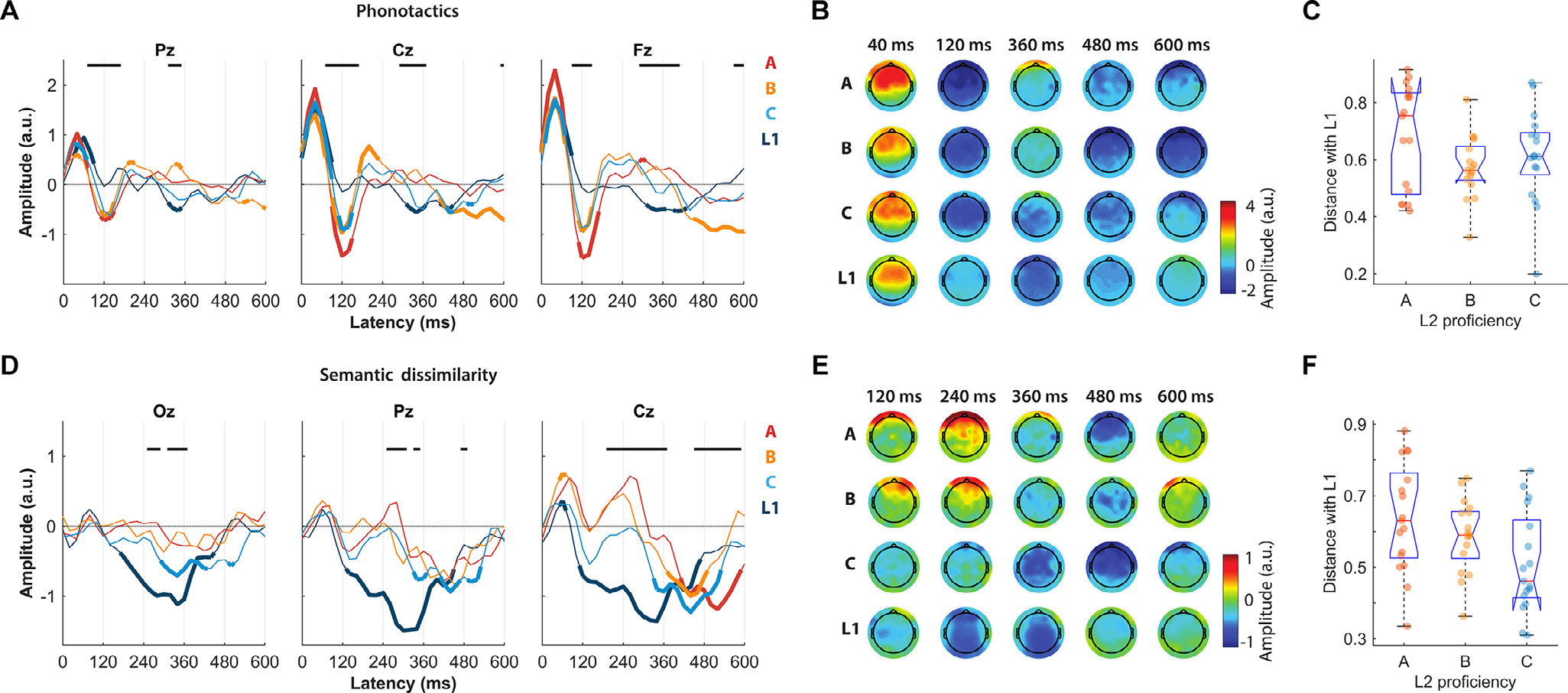
Effect of proficiency on the EEG responses to phonotactic and semantic dissimilarity regressors. (A) Model weights of the phonotactic TRF for three selected midline EEG channels at peristimulus time latencies from 0 to 600 ms. The results for distinct participant groups are color-coded. Thick lines indicate weights that were significantly different from zero across all subjects of a group (p < 0.05, Wilcoxon signed rank test, FDR corrected). Horizontal black lines indicate a significant effect of group proficiency on the TRF weights (point-by-point one-way ANOVA with FDR correction, p < 0.05). (B) Topographies of the phonotactic TRF weights for five selected time latencies. (C) Cosine distance of the phonotactic TRF for individual L2 participants with the average L1 TRF. The distance was calculated based on all electrodes and time latencies. (D) Model weights of the semantic dissimilarity TRF for selected EEG channels. (E) Topographies of the semantic dissimilarity TRF weights for five selected time latencies. (F) Cosine distance of the phonotactic TRF for individual L2 participants with an average L1 TRF.

TRF weights corresponding to the phonotactic descriptor (TRF_Pt_, a subset of TRF _ALL_) showed significant effects of latency (three-way ANOVA, *F* (28,6670) = 34.58, *p* < 10^−100^) and electrode

(*F* (4,6670) = 7.83, *p* < 2.7*10^−6^), a significant proficiency*latency interaction (*F* (56,6670) = 2.75, *p* = 6.6*10 ^−11^) and latency*electrode interaction (*F* (112,6670) = 3.66, *p* = 3.9*10^−34^), but *no effect of proficiency* (*F* (2,6670) = 2.44, *p* = 0.087) and no significant proficiency*electrode interaction (*F* (8,6670) = 0.25, *p* = 0.98). Fig. 3 A compares the corresponding TRF weights (part of TRF_ALL_) between proficiency groups at three scalp locations of interest. Qualitatively different TRF patterns emerged between groups, with an early positive component (~40 ms) that emerged consistently for all groups, an expected longer latency component (300–500 ms) that was less pronounced for L2 subjects than for L1 subjects but was significant for L2 with high and medium proficiency, and an unexpected earlier component (~120 ms) that emerged consistently only for all L2 groups but not the L1 group (FDR-corrected Wilcoxon test). The same latencies showed significant effects of proficiency group, which were measured as a point-by-point one-way ANOVA (*p* < 0.05, FDR corrected; Fig. 3 A reports the significance of that test, and its effect size is reported in Supplementary Fig. 5). The topographical patterns in Fig. 3 B further clarify that this effect of proficiency was distributed across most scalp areas, but especially in centro-frontal scalp areas at 120 ms, whereas the effect at a latency of approximately 360 ms showed centro-parietal patterns. We also studied the effect of proficiency on the cortical encoding of speech by assessing whether the TRF of L2 participants became more similar to that for L1 participants with proficiency. This distance was calculated with a cosine metric over all electrodes and over time latencies of the TRF simultaneously between each L2 participant and the average of all L1 subjects. The analysis did not indicate a significant effect of proficiency, i.e., there was no significant difference between the A, B, and C groups (Fig. 3 C: one-way ANOVA, *F* (1.4, 33.2) = 2.0; *p* = 0.14), suggesting that the effects of proficiency on L2 TRF should be studied for individual TRF components.

### 3.4. Stronger and earlier cortical responses to semantic dissimilarity with proficiency

A similar analysis was conducted based on semantic dissimilarity rather than on phonotactic scores. Specifically, content words were described according to a 400-dimensional feature space that was identified based on word co-occurrence (word2vec algorithm; Mikolov et al., 2013). Then, semantic dissimilarity was quantified as the *distance* of a word with the preceding semantic context, thus resulting in a vector marking the onset of all content words with these distance values (see Methods) (Broderick et al., 2018). Model weights corresponding to the semantic dissimilarity descriptor (TRF_Sem_, a subset of TRF_ALL_) showed *significant effects of proficiency* (three-way ANOVA, *F* (2,6670) = 14.9, *p* = 3.4*10^−7^) and latency (*F* (28,6670) = 38.50, *p* < 10 ^−100^), no significant effect of electrode (*F* (4,6670) = 0.22, *p* = 0.924), a significant proficiency*latency interaction (*F* (56,6670) = 2.87, *p* = 7.1*10^−12^) and latency*electrode interaction (*F* (112,6670) = 2.51, *p* = 4.5*10^−16^), and no significant proficiency*electrode interaction (*F* (8,6670) = 1.28, *p* = 0.24). Fig. 3 D shows TRF_Sem_ for three selected scalp channels. The average TRF_Sem_ for L1 participants was consistent with the results shown by Broderick and colleagues (Broderick et al., 2018), with a negative component peaking at peristimulus latencies of 340–380 ms. Similar TRF patterns emerged for the L2 C-level participants, whose average TRF_Sem_ values showed a negative component at comparable time latencies, with peak latencies between 340 and 440 ms (depending on the EEG channel). As expected, we observed significant effects of proficiency group over central and posterior scalp areas, which were measured as a point-by-point one-way ANOVA (*p* < 0.05, FDR corrected; Fig. 3D reports the significance of that test, and its effect size is reported in Supplementary Fig. 5). Interestingly, an unexpected significant bilateral centro-frontal negativity (BCN) peaking between 440 and 520 ms appeared in all L2 subjects but not in L1 subjects.

The latency of these negative components was assessed at an individual-subject level for the electrodes Oz, Pz, Cz, Fz, and Fpz by identifying the latency between 300 and 600 ms where the TRF has the largest negative magnitude. We found a significant effect of proficiency on trough latency (two-way ANOVA, *F* (3,312) = 4.22, *p* = 0.006). Furthermore, this effect was found to correspond to a negative correlation between peak latency and proficiency, which was strongest at the Cz location (Spearman’s correlation *ρ* = −0.37, *p* = 0.003). As for phonotactics (see previous section), we also assessed the cosine distance between the TRF of each L2 participant and the average TRF across all L1 participants, taking into consideration all TRF latencies and electrodes simultaneously. In this case, this distance showed a significant effect of proficiency (Fig. 3F: one-way ANOVA across the three proficiency levels, *F* (1.83, 42.1) = 3.7; *p* = 0.033), indicating a robust progressive L2-to-L1 convergence for semantic dissimilarity TRFs with proficiency.

### 3.5. Decoding language proficiency

**Fig. 4.**
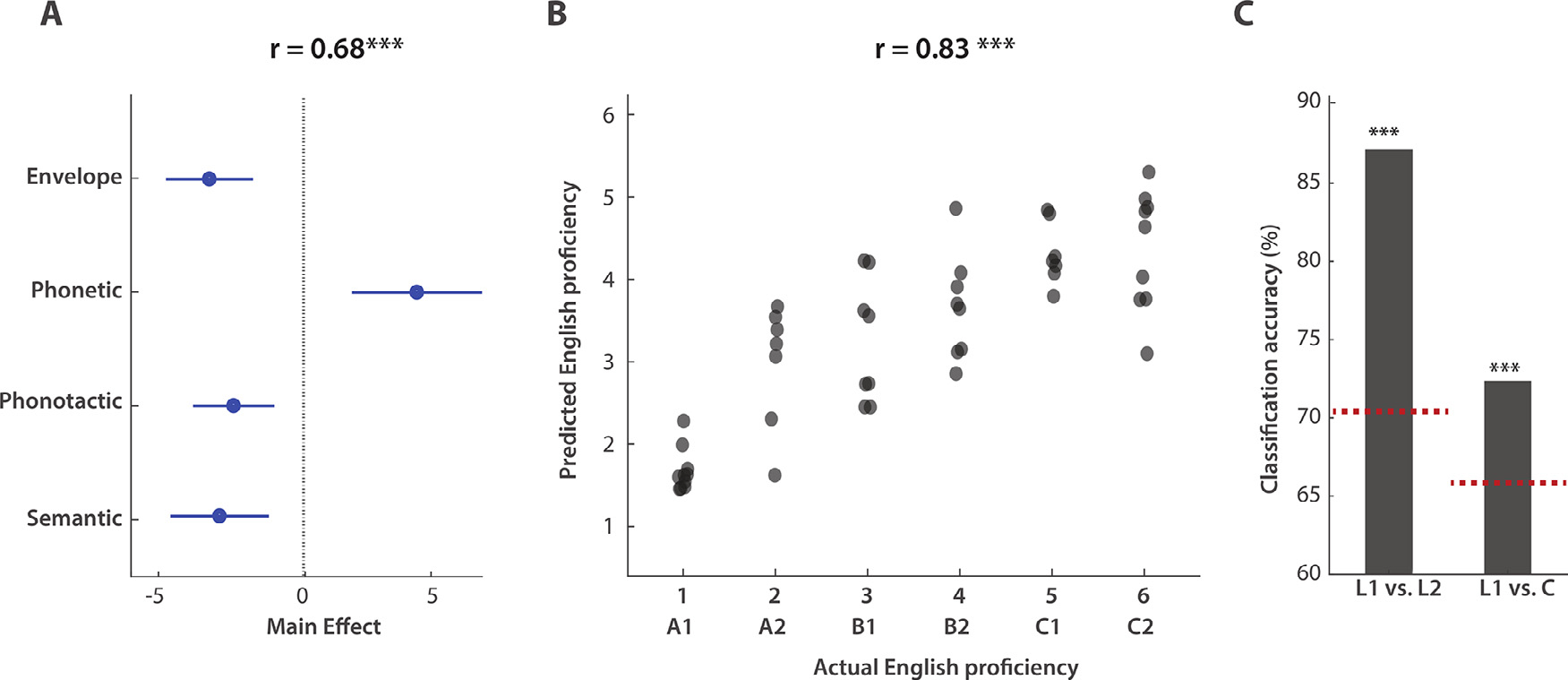
Accurate decoding of L2 proficiency from EEG data. (A) A multilinear principal component analysis (MPCA) was performed on the TRF weights corresponding to speech descriptors at all linguistic levels of interest. The first MPCA component was retained for the TRFs corresponding to Env, Phn, Pt, and Sem. The combination of these four features was predictive of L2 proficiency (r = 0.68), with significant effects for all features that were not due to group differences in age or attention. (B) A support-vector regression analysis shows that EEG data accurately predicted the L2 proficiency level at the individual subject level (r = 0.83, MSE = 1.14). (C) Classification accuracy for L1 versus L2 and L1 versus C-level L2. The red dotted lines indicate the baseline classification levels, which were calculated as the 95^th^ percentile of a distribution of classification accuracies derived after randomly shuffling the output class labels (N = 100). (For interpretation of the references to color in the text, the reader is referred to the web version of this article.)

Our results indicate that language proficiency modulates cortical responses at various linguistic processing levels. Given this relation, we examined the extent to which the proficiency of a subject could be predicted from the combined effects of different linguistic features. First, multilinear principal component analyses (MPCAs) were conducted on the TRF weights corresponding to Env, Phn, Pt, and Sem separately, and the first component was retained for each of them. In doing so, information spacing along three dimensions (EEG channels, time latencies, and stimulus features, e.g., phonetic features) was compressed into a single value for each participant. A linear regression model was then fit to predict L2 proficiency (L1 subjects were excluded from this analysis) based on the four aforementioned TRF features. Fig. 4 A shows the effect of each regressor on the model fit (coefficient estimate and standard error), with an overall regression correlation *r* = 0.68. Note that we are interested in the absolute value of the effect of each TRF feature, as the sign is arbitrary due to the MPCA step. Significant effects were measured for each of the four features, and this was true also when the ‘age’ information and the ‘one-back repetition detection’ score (which was a measure of the attentional engagement to the experiment) were included in the regression fit. This result confirmed that the main effect of proficiency was not due to attention or age.

A similar decoding approach was then used to assess whether and how robustly L2 proficiency could be decoded based on EEG indices of language processing. A set of 26 features was identified to describe the effects of L2 proficiency on the TRFs most comprehensively. Features were based either on the TRF weights (as in Fig. 4 A), on the EEG prediction correlations based on subject-specific TRF models, or on EEG prediction correlations for each subject when using average TRF models that were fit for the other subjects in A, B, C, and L1 groups separately (generic modeling approach; G.M. Di Liberto and Lalor, 2017; Jessen et al., 2019) (see Methods for a detailed list of features). Each of the 26 feature vectors had multiple dimensions (e.g., electrodes and time latency). For this reason, as described above, MPCA was used to reduce those vectors to one-dimensional regressors. Support vector machine (SVM) regression was used to decode L2 proficiency based on such regressors. A backward elimination procedure identified a reduced set of features (Supplementary Table 1) whose combination produced optimal L2 proficiency decoding scores, with *MSE* = 1.14 and Pearson’s correlation *r* = 0.83, *p* = 3*10^−13^ (Fig. 4 B). Another way to quantify the quality of the proficiency decoding is to assess the A- vs. C-level classification by placing a simple threshold on the prediction values (a value of 3.5, which cuts the prediction space in half). This binary classification could identify A- vs. C-level participants with 91% accuracy.

Further analyses were conducted to assess the effect of “nativeness ” on the EEG responses to speech. Specifically, differences in language processing between L1 and L2 subjects may be in part driven by a fundamental distinction between native and nonnative language processing that is not due to proficiency *per se* but rather due to differences in the L1 and L2 processing networks (Cao et al., 2013; Morgan-Short et al., 2012). In fact, the TRF results in Figs. 1–3 indicated that higher proficiency levels do not always lead to EEG responses that are equivalent to those of native speakers. Specifically, though there was some level of L1-L2 convergence for phoneme-level TRFs, this phenomenon was less pronounced for phonotactic and semantic dissimilarity responses, with marked differences between L1 and C-level L2 (e.g., the latency of the negative component at ~120 ms in TRF_Pt_). Here, we attempted to disentangle those differences from the effect of L2 proficiency by conducting an SVM binary classification analysis for L1 versus L2 participants. This procedure used the same 26 features and backward elimination strategy as in the previous regression analysis. First, an L1 versus L2 classification accuracy of 87% was obtained when all 71 subjects were included in the analysis, with a baseline classification accuracy of 70% (95^th^ percentile of a distribution of classification accuracy values when L1-L2 labels were randomly shuffled – 100 shuffles). To reduce the contribution of proficiency to the classification result, the same analysis was performed on L1 and C-level L2 participants only. In this case, a classification accuracy of 73% was measured, with a baseline of 66%, thus suggesting that the EEG responses to continuous speech reflect both the influence of L2 proficiency and nativeness. Nevertheless, it is important to highlight that this result emerged in a small cohort of L1 and L2 participants. Furthermore, different behavioral tasks were used for L1 and L2 participants, as the L2 group included subjects who could not understand the speech. As such, further work with a more specific design and a larger sample size is needed to confirm this result.

## 4. Discussion

The human brain responds differently when listening to a second-language compared to the responses when listening to native speech (Hanna et al., 2016; Ojima et al., 2005; White et al., 2012). Typically, lower listening performance is noted in the former that varies between individuals and can be quantified with standardized language tests. Despite the general consensus for the cognitive, social, and economic advantages that come with high L2 proficiency, the neural underpinnings of second-language perception and learning remain unclear (Chang, 2019; Flege, 1995). One reason that this issue remains unresolved is methodological. Experimental evidence derived from direct neural measures is minimal and often limited to single linguistic properties (Brandmeyer et al., 2013; Khachatryan et al., 2016; Kimppa et al., 2019; Mueller, 2005; Osterhout et al., 2008), thus offering only a partial view of this complex brain mechanism. The present study established a methodological framework to provide a more comprehensive examination of the language processing system in naturalistic conditions. We isolated neural indices of speech perception at multiple processing stages from EEG responses to natural speech, revealing marked effects of L2 proficiency that were robust at the individual subject level. Overall, the results confirmed our hypothesis that the cortical encoding of speech in L2 listeners changes with proficiency and that EEG responses to natural speech are sensitive to its change for distinct linguistic properties, even at the level of individual phonemic contrasts.

Previous studies that investigated L2 perception in naturalistic paradigms focused on the relationship between neural activity and the acoustic envelope and found stronger coupling in L2 subjects than L1 subjects (Song and Iverson, 2018). That EEG result, which was found using a selective attention listening task in a multitalker scenario, pointed to a link between increased listening effort and stronger cortical tracking of the speech envelope. However, it remains unclear which of the linguistic and nonlinguistic properties of speech correlated with the acoustic envelope results in this increased cortical tracking. In fact, an increased coupling between EEG data and the speech envelope could reflect increased encoding of acoustic features, stronger reliance on higher order processes, or even activation of distinct cortical areas. For example, recent work indicated that envelope tracking increases with age due to a stronger engagement of higher order areas, thus reflecting a difference in processing strategy for older listeners (Brodbeck et al., 2018b; Presacco et al., 2019). Here, the shape of the envelope TRF significantly contributed to L2 proficiency decoding (Fig. 4), even though the modulation was not specific to any individual response component, which is in line with a link between acoustic-level encoding and effort. Interestingly, this result was obtained using a single-talker task with no competing noise. Using this same approach with a more cognitively demanding task (Hjortkjaer et al., 2018; O’Sullivan et al., 2014) could help tease apart the effects of L2 proficiency and listening effort on the cortical encoding of acoustic features.

As we had hypothesized, the cortical encoding of phonemes changed with L2 proficiency, becoming progressively more similar to that of L1, which is in line with perceptual theories such as the expanded Native Language Magnet Theory (NML-e; Kuhl et al., 2008) and the Perceptual Assimilation Model (PAM-L2; Best and Tyler, 2007). A core principle of these theories is that adults perceive unfamiliar phonemes according to some similarity to phonemes that are present in their existing (native) phonemic inventory. This existing inventory, or phoneme map, would explain the facility in detecting native sounds and the difficulty in detecting and discriminating new phonemes. Our data are in line with this view and provide important empirical evidence of such an internal phonological map and of how it changes across various L2 proficiency levels. Our TRF analysis has discerned individual phonemic contrasts, showing that the cortical encoding of phonemes becomes progressively more sensitive to contrasts existing in English but not Standard Chinese (Fig. 2). This work extends previous findings on the cortical encoding of phonemes (Daube et al., 2019; Di Liberto et al., 2015; Khalighinejad et al., 2017) by demonstrating that EEG responses to natural speech show sensitivity to individual phoneme contrasts with response patterns that become progressively more categorical with proficiency. Furthermore, the aforementioned result goes beyond previous work (Daube et al., 2019) by revealing a low-frequency EEG component that could not be explained by simple acoustic features such as the acoustic envelope, the derivative of the envelope, and spectrogram attributes. Our results are in line with the majority of the theories on L2 perception, which suggest the impact of a subject’s L1 on L2 phonological encoding. Specifically, Fig. 2A indicates that the native language constitutes a “starting point ” for phonological encoding of L2 sounds in L2 learners, which then changes with experience and converges toward the encoding for L1 listeners.

Reproducing this work on participants with other native languages could provide us with detailed insights into the effect of the native language on phoneme encoding in high-proficiency L2 learners. In fact, the languages of interest in the present study were English and Mandarin, with profound differences involving all the hierarchical levels investigated. Indeed, we expect languages with higher overlap in, for example, the phonological inventory to show a strong effect of proficiency on the properties that have to be learned and a weaker effect on phonological processing. Further studies with larger sample sizes could use this approach to study the effect of different learning methodologies on L2 perception at different stages of learning. Finally, additional data with a balanced design, in which subjects listen to both their native language and a nonnative language, could reveal whether and how learning a particular L2 influences the cortical processing of the native language (Chang and Mishler, 2012; Zinszer et al., 2015), as was postulated by the bidirectional cross-linguistic influence principle in the Speech Learning Model (SLM; Flege, 1987).

Proficiency was also shown to shape language encoding at the phonotactic level, with TRFs in L2 subjects progressively converging toward L1 TRFs. Our results indicate two effects of phonotactics. First, we measured a TRF component peak at speech EEG latencies of approximately 300–450 ms, which we had hypothesized as it was measured in a previous EEG study by our group (Di Liberto et al., 2019), with more negative responses for higher proficiency levels (Fig. 3A). Second, an effect at shorter latencies of approximately 120 ms, in which a negative component that was not present for L1 participants emerged for L2 participants. Interestingly, a component reflecting phonotactics was previously measured at that speech-neural signal latency using MEG (Brodbeck et al., 2018a) but not using EEG. Our finding provides a new link between the EEG and MEG literature by clarifying that phonotactic features modulate EEG responses at both shorter and longer latencies and that the effect at shorter latencies emerges for L2 learners but not native speakers. This discrepancy may be due to the difference in the type of signal recorded by EEG and MEG modalities. The larger values for users with lower proficiency could reflect an effect of surprise on the phoneme sequences due to the use of an incorrect (or imperfect) model of phonotactics.

Semantic dissimilarity TRFs were previously shown to be characterized by a negative centro-parietal component at speech-EEG latencies of approximately 350–400 ms. This finding is in line with previous work on the N400 (Borovsky et al., 2012; Kutas and Federmeier, 2011; Kutas and Hillyard, 1980), an event-related potential component that is modulated by intelligibility and attention and that has the same spatiotemporal characteristics (Broderick et al., 2018). Similarly, we expected strong response negativity for users with higher language proficiency and no response for people with no English proficiency at all (note that negative TRF values correspond to negative voltage values of the impulse response of Sem). Consistent with this hypothesis, our results identified a posterior component with magnitude that increases with proficiency (Fig. 3 D). In addition, an unexpected centro-frontal component arose at latencies of approximately 440–520 ms, which was negatively correlated with the latency of response rather than the magnitude of the component. This bilateral centro-frontal negativity (BCN) emerged even for participants with no English understanding, thus reflecting neural correlates time-locked to word onset but not semantics *per se*. This component may instead be related to other processes, such as sentence structure processing, memory tasks, and the process of learning frequent words (Chen et al., 2014; Coulson and Kutas, 2001; Manfredi et al., 2018; Wlotko and Federmeier, 2012). Further work is needed to clarify whether that signal reflects, for example, the familiarity with particular words, or whether it is related to ERP components such as the left anterior negativity (LAN), which was shown to reflect processing difficulties in morpho-syntax (Meltzer and Braun, 2013; Steinhauer and Drury, 2012).

Although both phonotactics and semantic level TRFs for L2 showed some level of convergence to L1, there was also a pronounced difference between L1 and C-level L2 participants, which was also reflected in the significant L1 versus C classification result in Fig. 4 C. This effect may reflect fundamental differences in the cortical mechanisms underlying L1 and L2 processing, rather than an effect of proficiency *per se*. This effect of nativeness that is somewhat different from the effect of proficiency is in line with the observation that a second language learned after a certain critical (or sensitive) period usually leads to lower language proficiency than that of a native speaker (Bialystok and Hakuta, 1999; Hartshorne et al., 2018; Purves et al., 2003). More data could provide further insights on this topic, for example, by comparing L1 monolinguals with bilinguals and multilinguals with a wide range of learning-onsets for the English language. Our results show different progressions for the L2-to-L1 convergence for distinct features. As we had hypothesized, such differences may be explained by the particular language-learning strategy adopted for distinct features. Further work targeting different second language learning strategies could contribute to revealing how strongly the linguistic encoding progression for distinct features is related to the particular learning approach. Further research is also needed to better understand the effect of nativeness, e.g., by comparing L1 and high-proficiency L2 listeners using a semantic task that guarantees the same level of comprehension for all participants. Such a task could not be employed in the present study, as the primary focus of this study was the effect of proficiency across A- to C-level L2 participants, which, by design, presented variable levels of comprehension.

Our analysis focused on just a few components of the speech processing hierarchy, namely, the acoustic, phonemic, phonotactic, and semantic levels. One powerful element of this framework is that it can be extended to other levels of processing without the need for additional data. In fact, the EEG responses to natural speech likely reflect many more components of interest than the ones targeted in this occasion; isolating these components following the model presented in this study would give us insights into each newly added feature and its link with proficiency, as well as provide us the chance to improve the accuracy of our EEG-based L2 proficiency assessment. For these reasons, we believe that a wide collaborative effort under a common protocol of data acquisition with EEG/MEG and natural stimuli could significantly and quickly advance our understanding of the speech and language cortical processing network (and could indeed extend to other questions of interest). Such an initiative would allow for the extension of our findings to other combinations of L1-L2 languages, as well as prompt the investigation of questions that require larger sample sizes. One such question concerns the opportunity for decoding multiple robust objective indices of proficiency from the same EEG recording. In fact, while the present investigation focused on coarsely grouping subjects into A, B, and C levels, such proficiency levels summarize a number of skills (e.g., vocabulary and phonological skills) that can change between participants within the same proficiency group; this aspect could represent important objective markers of language proficiency.

Understanding the neural underpinnings of second language perception and learning becomes particularly relevant when we consider that there are more children throughout the world that have been educated via a second (or a later acquired) language rather than exclusively via their L1 (Tucker, 2001). Furthermore, there is evidence for the perceptual advantage of bilinguals and multilinguals that is due to cross-language transfer (Chang, 2012; Chang and Mishler, 2012; Kartushina and Frauenfelder, 2014), and particular combinations of languages may be better than others in the emergence of such a benefit. Further work in this direction may provide us with tools to predict the perceptual advantage that a particular second language would bring to a person given their background; this work could constitute the basis for a procedure that, for example, could inform us on which second languages should be encouraged in school for particular individuals. Indeed, additional work is needed to assess the parameters, making this methodology applicable in particular cohorts of participants. While recent work has partly answered this question, indicating that EEG indices of acoustic-phonetic (Di Liberto et al., 2018b; Di Liberto and Lalor, 2017; Jessen et al., 2019) and semantic-level (Broderick et al., 2020) perception can be isolated with ~10 minutes of EEG data in infants, children, and older participants, as well as in hearing-impaired individuals (Decruy et al., 2020; Fuglsang et al., 2020), more work is needed to evaluate the reliability of the ensemble of EEG indices discussed in the present study.

## Supplementary Material

Supplementary materials
