# Supplementary material for: Neural representation of linguistic feature hierarchy reflects second-language proficiency
Source: Neuroimage. Author manuscript; Available in PMC 2021 Oct 20. (PMC8527895; doi:10.1016/j.neuroimage.2020.117586)
Supplement: Supplementary materials [file NIHMS1731879-supplement-Supplementary_materials.pdf]

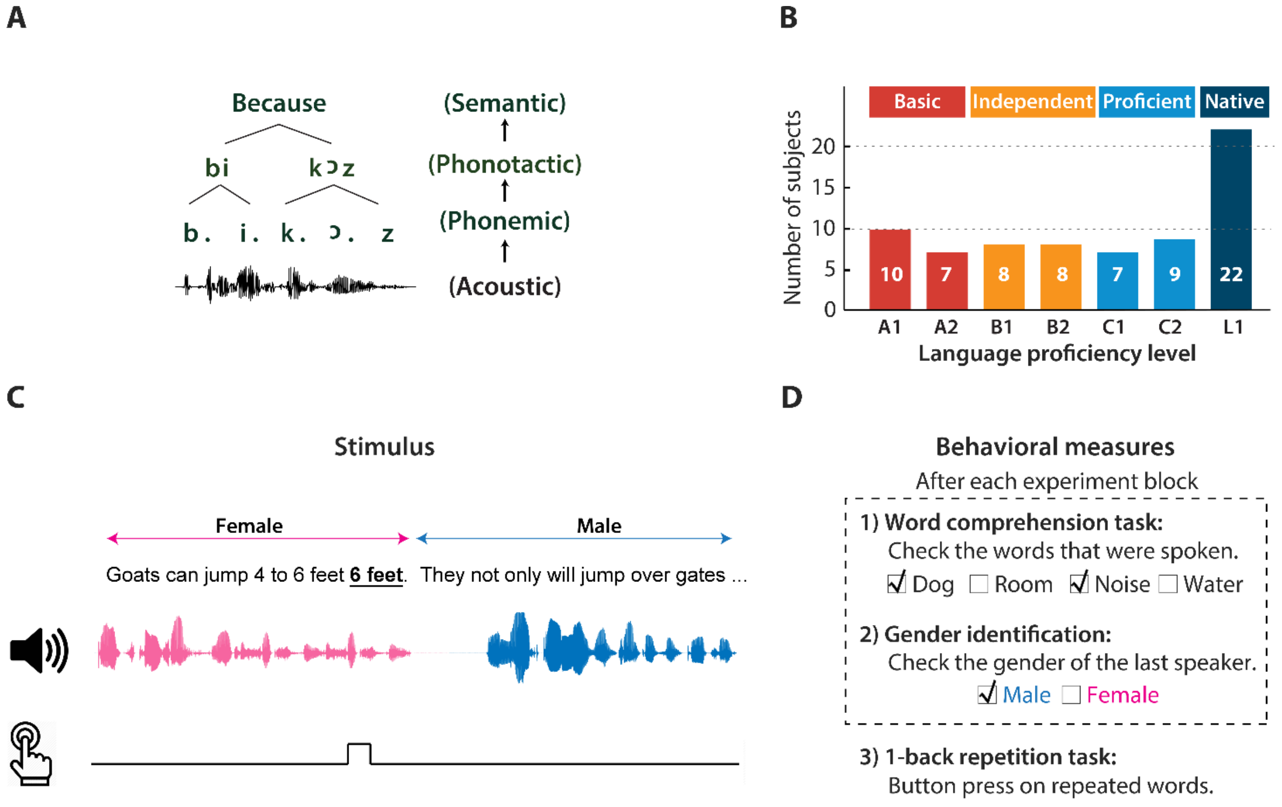

Supplementary Figure 1. *Experimental setup and behavioral results. (A) Selected part of the speech processing hierarchy, from low-level acoustics (bottom) to higher-level linguistic properties (top). (B) Demographic distribution of the proficiency levels. (C) Sentences spoken by a male speaker and a female speaker were presented in alternation. Participants were asked to detect a one-back phrase repetition (2-4 words), which occurred 1-5 times per experimental block, by pressing a button during the experimental block. (D) After each block, participants were asked to identify words that were spoken during the block from a list of eight and to indicate the gender of the speaker at the end of the block.*

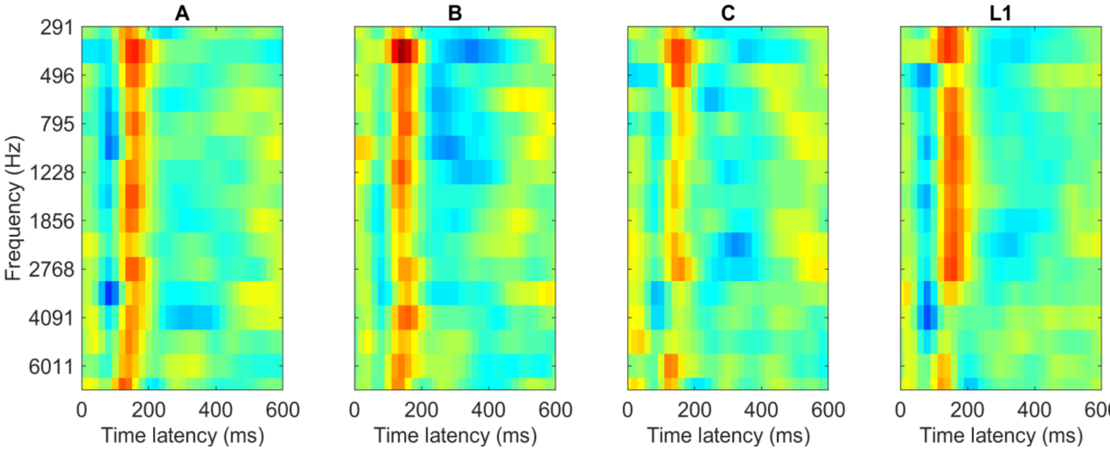

Supplementary Figure 2. *Average TRF$_{sgr}$ weights across subjects at electrode Cz and peristimulus time latencies from 0 to 600 ms for each proficiency level.*

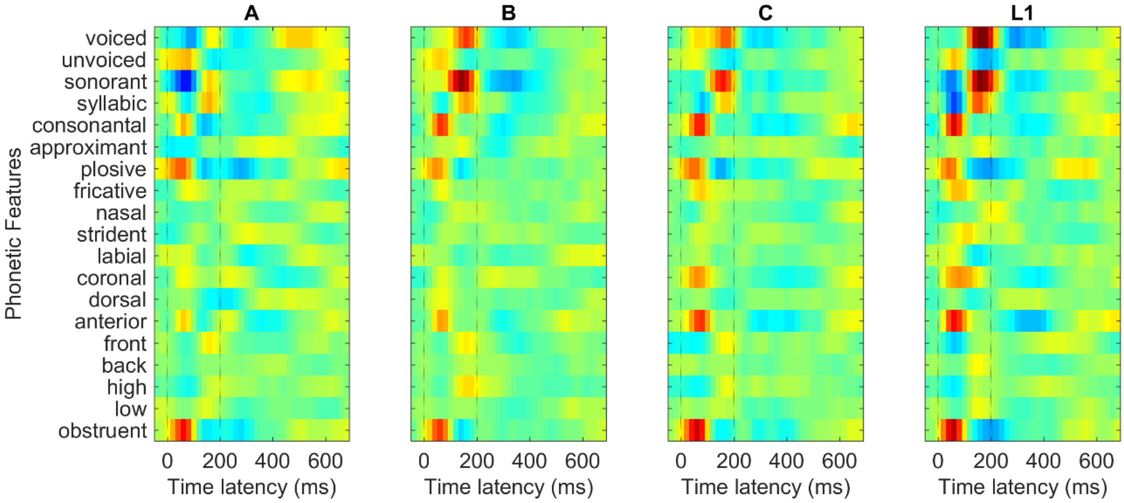

Supplementary Figure 3. *Average TRF$_{phn}$ weights across subjects at electrode Cz and peristimulus time latencies from 0 to 600 ms for each proficiency level. These TRF weights were used to produce the results in Figure 2A.*

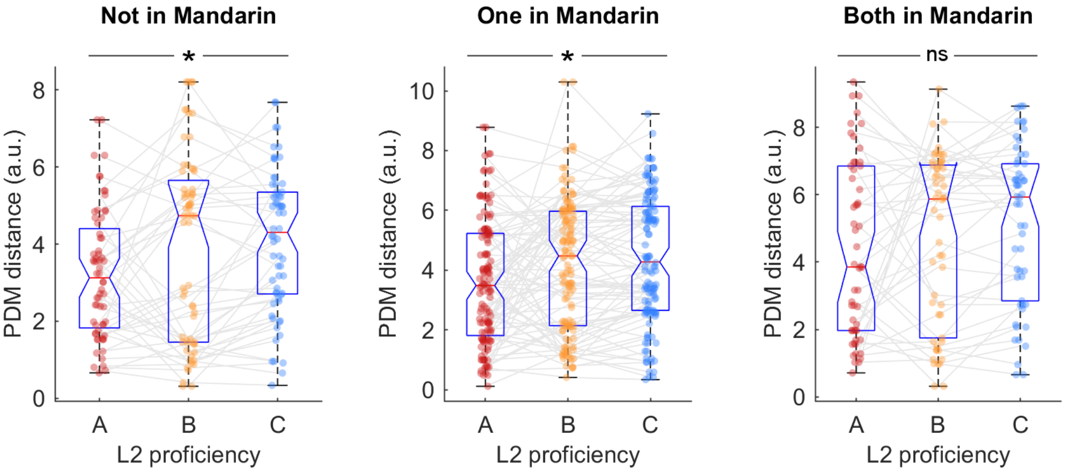

Supplementary Figure 4. *Distance of all English phoneme contrasts in the phoneme distance map (PDM) grouped by the L2 proficiency group. (Left) PDM distances for contrasts in which both phonemes did not belong to the Mandarin inventory – ANOVA $F(1.6, 150.3) = 3.8$, $p = 0.024$. (Middle) PDM distances for*

contrasts in which only one of the two phonemes belonged to the Mandarin inventory – ANOVA F(1.4, 245.8) = 4.4, p = 0.013. (Right) PDM distances for contrasts that exist in Mandarin – ANOVA F(1.26, 90.9) = 0.9, p = 0.39.

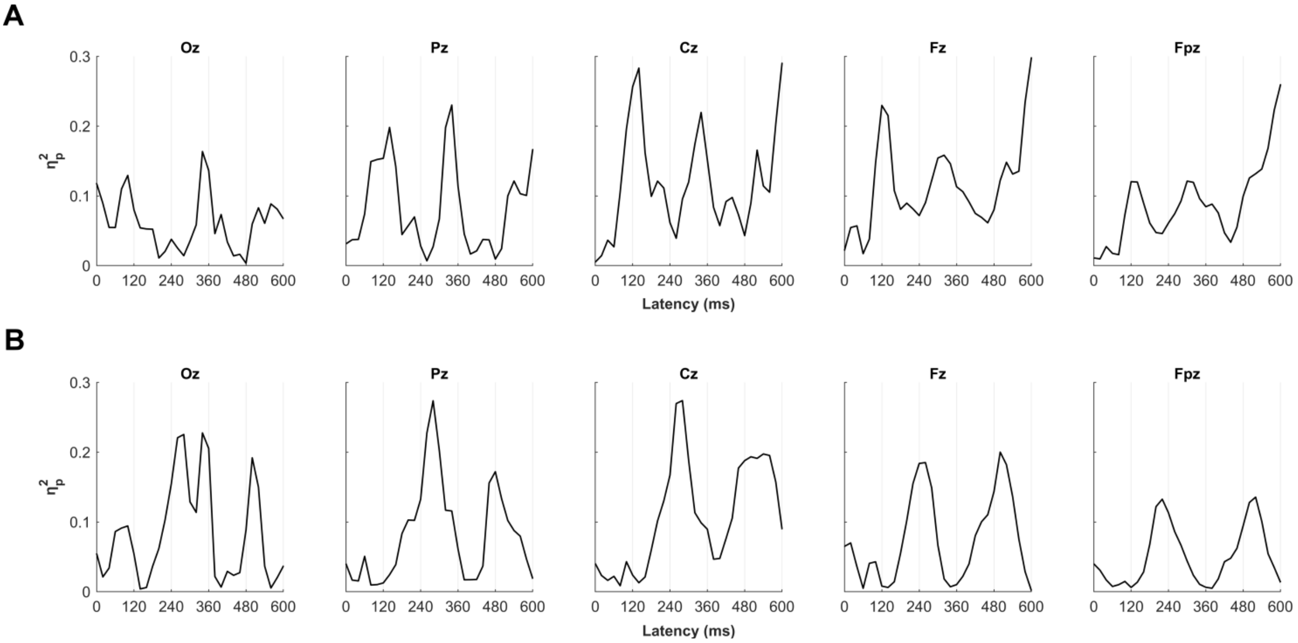

Supplementary Figure 5. *A point-by-point one-way ANOVA was performed on individual electrodes and time latencies to assess the effect of proficiency on the TRF weights for Pt (A) and Sem (B). This figure reports the effect size of such tests (partial eta-squared, η^2_p).*

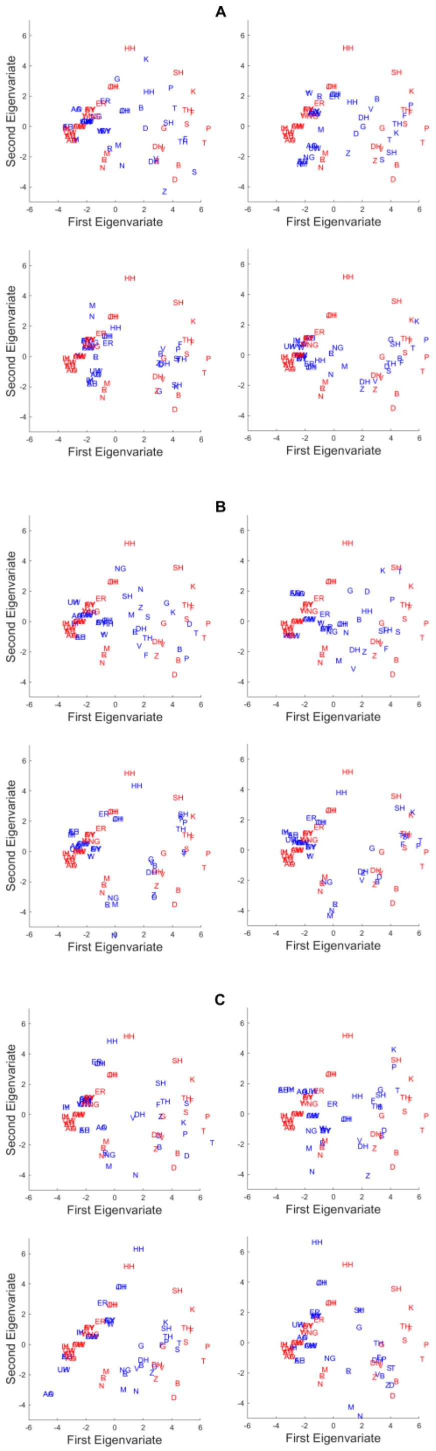

Supplementary Figure 6. *Phoneme distance maps (PDM) for selected individual L2 subjects with A, B, and C levels of proficiency (blue) compared with the PDM for all L1 subjects.*

				Optimal decoding feature for:		Explained variance by the first PCA component across:	
		Feature	Description	L2	L1 vs. L2	EEG Channels	TRF time-lags
TRF weights		Env	Temporal dimension of the spectrogram response			0.68	0.24
		Sgr	Frequency dimension of the spectrogram response			0.61	No time-lags
		Phn	Temporal and feature dimensions of the phoneme response			0.14	0.54
		Po	Temporal dimension of the phoneme onset response			0.62	0.2
		Pt	Temporal and feature dimensions of the phoneme response			0.44	0.27
		Sem	All EEG channels and time-lags			0.58	0.34
		Sem, Front-Back	Pz minus FPz for time-lags between 300 and 600 ms			Selected channels	No time-lags
		Pt, short latencies	Phonotactics for time-lags between 80 and 140 ms				
		Pt, longer latencies	Phonotactics for time-lags between 300 and 700 ms				
EEG prediction correlations	SubjectSpecific	Env	Prediction r when using Env feature			Average of all electrodes	No time-lags
		Phn	Prediction r when using Phn feature				
		Pt	Prediction r when using Pt feature				
		Sem	Prediction r when using Sd feature				
		PhnEnv'Sgr - Env'Sgr	Increase in Prediction r when using Phn features				
	Generic	Envelope: generic A	Prediction r when using the Env generic model fit on the A group			0.63	No time-lags
		Envelope: generic B	Prediction r when using the Env generic model fit on the B group			0.68	
		Envelope: generic C	Prediction r when using the Env generic model fit on the C group			0.68	
		Envelope: generic L1	Prediction r when using the Env generic model fit on the L1 group			0.61	
		PhnEnv'Sgr - Env'Sgr: generic A	Increase in prediction r when using Phn - generic model A group			0.23	
		PhnEnv'Sgr - Env'Sgr: generic B	Increase in prediction r when using Phn - generic model B group			0.42	
		PhnEnv'Sgr - Env'Sgr: generic C	Increase in prediction r when using Phn - generic model C group			0.5	
		PhnEnv'Sgr - Env'Sgr: generic L1	Increase in prediction r when using Phn - generic model L1 group			0.48	
		Sd: generic A	Prediction r when using the Sd generic model fit on the A group			0.41	
		Sd: generic B	Prediction r when using the Sd generic model fit on the B group			0.46	
		Sd: generic C	Prediction r when using the Sd generic model fit on the C group			0.45	
		Sd: generic L1	Prediction r when using the Sd generic model fit on the L1 group			0.57	

Supplementary Table 1. *The 26 features used for the proficiency decoding and L1* vs. *L2 classification analyses. Features are grouped into TRF weights, EEG prediction correlations when using subject-specific models, and EEG prediction correlations when using generic models that were averaged within a particular proficiency group. A backward elimination procedure was used for feature selection. Features that were selected for decoding are indicated with a colored cell. Several features were the first component resulting from an MPCA across EEG channels, TRF time lags, or both. The table reports the explained variance for the first component in which MPCA was used.*

	Place of Articulation						
	Bilabial	Labio-dental	Dental	Alveolar	Post-alveolar	Velar	Glottal
**Manner of Articulation**							
Stop	P B			T D		K G	
Fricative		F V	TH DH	S Z	SH ZH		HH
Affricate					CH JH		
Nasal	M			N		NG	
Lateral approximant				L			
Approximant	W				R	Y	

Phoneme exists in English & Mandarin

Phoneme exists in English only

Supplementary Table 2. *Manner of articulation and place of articulation features for the English consonants analyzed in Figure 2. Phonemes are colored to indicate which ones exist in both English and Mandarin.*
